# Exploiting Direct Laser Writing for Hydrogel Integration into Fragile Microelectromechanical Systems

**DOI:** 10.3390/s19112494

**Published:** 2019-05-31

**Authors:** Julian Menges, Steffen Klingel, Egbert Oesterschulze, Hans-Jörg Bart

**Affiliations:** 1 Department of Mechanical and Process Engineering, Chair of Separation Science and Technology, TU Kaiserslautern, 67663 Kaiserslautern, Germany; 2 Department of Physics, Physics and Technology of Nanostructures, Nano Structuring Center, TU Kaiserslautern, 67663 Kaiserslautern, Germany; klingel@rhrk.uni-kl.de (S.K.); oester@rhrk.uni-kl.de (E.O.)

**Keywords:** direct laser writing, multiphoton lithography, hydrogel microstructuring, soft materials, responsive hydrogels, liquid sensing

## Abstract

The integration of chemo-responsive hydrogels into fragile microelectromechanical systems (MEMS) with reflective surfaces in the micron to submicron range is presented. Direct laser writing (DLW) for 3D microstructuring of chemoresponsive “smart” hydrogels on sensitive microstructures is demonstrated and discussed in detail, by production of thin hydrogel layers and discs with a controllable lateral size of 2 to 5 µm and a thickness of some hundred nm. Screening results of polymerizing laser settings for precision microstructuring were determined by controlling crosslinking and limiting active chain diffusion during polymerization with macromers. Macromers are linear polymers with a tunable amount of multifunctional crosslinker moieties, giving access to a broad range of different responsive hydrogels. To demonstrate integration into fragile MEMS, the gel was deposited by DLW onto a resonator with a 200 nm thick sensing plate with high precision. To demonstrate the applicability for sensors, proof of concept measurements were performed. The polymer composition was optimized to produce thin reproducible layers and the feasibility of 3D structures with the same approach is demonstrated.

## 1. Introduction

Smart sensitive hydrogels are crosslinked polymers, whose mass reversibly changes in response to chemical or physical stimuli of the surrounding solvent. The mass change is commonly induced by swelling as a response of changes in pH [1,2,3,4,5,6,7,8], temperature [9], ionic strength [10,11], irradiation [12] or target molecules [13,14,15,16,17]. These properties make them interesting for a variety of applications like chemical or biosensing [18,19] as well as actuators [20]. Since diffusion of solvents and solutes into thin layers or structures is fast [20], the integration into microelectromechanical systems (MEMS) is of particular interest as it offers fast, ultra-sensitive and parallel chemical sensing in small volumes. This enables integration into microfluidic or lab-on-a-chip devices rendering it promising for on-demand clinical diagnostics. To implement hydrogel microstructures, there are three basic methods known in the literature [21]: Spin-coating of monomer solutions followed by spatially controlled photopolymerization (photolithography) [22], soft lithography (replica molding) [23,24] and dry etching of thin hydrogel layers using metal or photoresist masks [25,26]. Each method has its limits and benefits in terms of resolution or feasibility and none of the methods can be used for precision structuring on fragile MEMS structures due to the necessary hard contact.

Direct laser writing (DLW) offers a promising solution to these challenges. In general, a femtosecond pulsed laser beam is focused into a negative-tone photo resist. In the gel, the incorporated energy gets absorbed by photo initiator molecules via two-photon absorption within the very focal volume, starting a polymerization. Relative movements of resist and laser focus allow for fabricating 3D structures of almost any desirable shapes [27,28]. Several hydrogels have been structured in this way, mostly aiming for biological compatibility [29,30,31,32,33], but relatively little is said in respect to precision structuring of responsive gels in MEMS [29,34,35]. In this article, a generic approach to structure responsive hydrogels on reflective surfaces by DLW and their embedding in MEMS is demonstrated.

**Figure 1:**
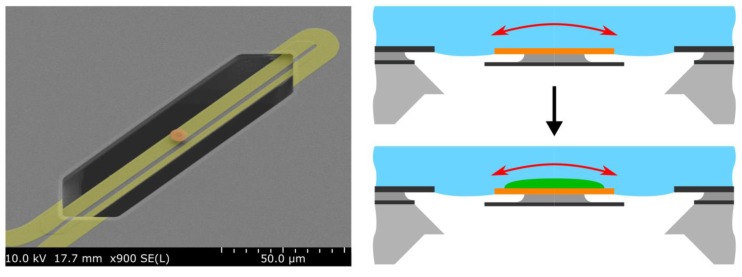
**Left**: False colored scanning electron microscopy image of the hybrid bridge microresonator [36]. **Right** top: Schematic cut view through the resonator bridge (black) and sensor plate (colored orange) single sided wetted by water (blue). Basic torsional mode of the bridge indicated by red arrow. Right bottom: Functionalization by hydrogel (green).

For mass sensing applications in liquids, we have invented the concept of partially wetted microresonators [36] to reduce, in particular, viscous losses during operation that have been a common issue [37] (Figure 1). Optical read-out of the microresonators vibration frequency was achieved with a conventional optical beam deflection technique. Driving an alternating current through the half-loop conductor integrated onto the resonator in a static external magnetic field allowed the actuation of the basic torsional mode. Finally, a network analyzer (Agilent 4395A) was used to feed the actuation frequency to the resonator and additionally monitor the resulting oscillation response [38]. Here, we make use of the sensor to demonstrate integration of a responsive hydrogel onto the plate and that it maintains its chemical properties by using the functionalized microresonator as a chemoselective sensor (Figure 1, right bottom).

When using DLW for hydrogels, there are multiple challenges. Polymerization is performed by radical initiation followed by chain growth. As long as the chains are short, they are thought to diffuse from the initiation area [39]. Depending on their mobility, this can result in either broadened or labile polymerization voxels or, in the worst case, no voxels. Additionally, a low level of crosslinking is necessary for many responsive hydrogels, which is in contrast to common DLW photoresist compositions, since high crosslinking does not allow for high swelling. Therefore, as an approach that makes use of large monomers with controllable crosslinking, a protocol based on linear polymer chains with polymerizable side groups called macromers [40] was developed. This way, crosslinking density and viscosity can simultaneously be defined by the macromer composition and additional monomers.

## 2. Materials and Methods

**Figure 2:**
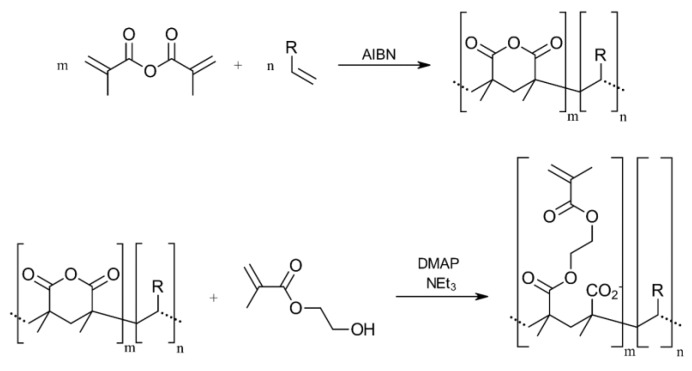
Synthesis of reactive MAAA- copolymer and subsequent hydrolysis with HEMA to receive a crosslinker (298 K).

All chemicals were received from Sigma Aldrich. Methacrylic acid (MAA), Triethylamine (NEt_3_), mono-2-(methacryloyloxy)ethyl succinate (MMAES) and 4-(dimethylamino)pyridine (DMAP) were received in >99%, hydroxyethyl methacrylate (HEMA) and 2,2′-azobis(2-methylpropionitrile) (AIBN) in 98%, methacrylic acid anhydride (MAAA) in 94% and N-isopropyl acrylamide 97% (NIPA) quality. Monomers contained inhibitors for stabilization. For macromer synthesis, inhibitors were removed by distillation in vacuo. In the resin solution, the inhibitor remained as received to guarantee stable samples until processing. For synthesis, MAAA was polymerized with other monomers of choice in acetone by addition of AIBN (Figure 2 top). At low concentrations ring closing instead of crosslinking is favored [41]. Next, anhydride is hydrolyzed with an excess of HEMA with DMAP as a catalyst and trimethylamine as a base (Figure 2 bottom) so precipitated polymer redissolves due to hydrolysis of the crosslinking MAAA.

Hydroxyl and amino groups undergo a reaction with MAAA and must therefore be avoided as monomer functionalities. MAA and acrylates such as NIPA were added as suitable monomers to tune macromer solubility. The product mixture was precipitated by addition of 1% HCl. M1 was designed for high crosslinking and M2 for pH responsive gels (Table 1). For purification the macromers were dissolved in acetone with 5% methyl alcohol and precipitated with diethyl ether three times.

The macromers were dissolved in a mixture of monomers and solvent to give solutions of usable viscosity for deposition (Table 2). For initiation, the photoinitiator phenylbis (2,4,6-trimethylbenzoyl)phosphine oxide (BAPO) was used, as it can undergo two-photon absorption [42]. The resin R1 was synthesized for the preliminary experiments, R2 for the resonator experiments and R3 for the “dry” structuring. The solvent dimethyl sulfoxide (DMSO) and an acidic large monomer (MMAES) were used for R3.

**Figure 3:**
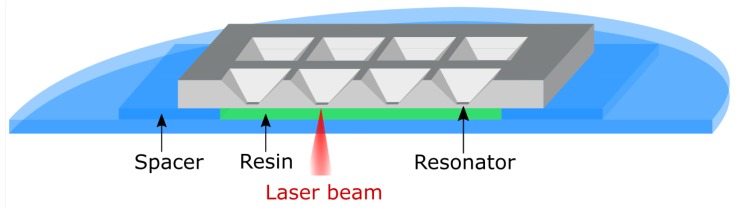
Assembly for direct laser writing (DLW). The chips (grey) were placed on a cover glass (blue) covered with a drop of resin solution (green), separated by glass cutouts. Illumination was done upside down through an oil immersion objective by the laser (red).

The resonators were on chips of 1 cm^2^ size and their surface consisted of a 200 nm reflective chromium layer. The samples were placed on a cover glass (BK7, 30 mm diameter, Menzel) covered with a drop of resin solution (Figure 3). Glass cutouts were used as spacers for reproducible distance and alignment. For DLW operation, a commercial system (Photonic Professional GT, Nanoscribe GmbH, Germany; 25× oil-objective, NA = 0.8) was used. A 780 nm pulsed femtosecond fiber laser was focused onto the resonator plate, exposing one position 20,000 times each for t_dwell_ = 0.02 ms, giving 0.4 s exposure time in total. This pulsed exposure over prolonged time is a necessity arising from the destructive interference of the reflected laser with the incoming beam close to the surface. This process allows for diffusion of the active polymerizing chains to the surface. Many single exposure shots (20,000 were empirically found to be a good compromise between quality and time) are necessary to utilize the proximity effects, facilitating the attachment of the structure onto the resonator surface. The power was set as a relative value P_rel_, where P_rel_ = 1 is the maximum power. The manufacturer specified the maximum laser power output at 100 mW with some losses in the optical system due to Fresnel reflections, absorption and scattering. For development, the glass-sample assembly was submerged in DMSO until the chip was released from the glass by itself. Then, the glass was removed and the chip was developed for 15 min followed by development in 1% solution of triethylamine in de-ionized water for 5 min. After rinsing with acetone and isopropanol, the samples were dried in a nitrogen stream.

Our aim was to structure pH responsive hydrogels, meaning a low level of crosslinking was necessary. The resulting macromers with a considerably increased amount of MAA are not soluble in the resin solutions but instead gel strongly. When using pure NIPAA macromers, the quality and reproducibility dropped significantly. Therefore, the macromer M2 was developed as it offered high solubility and high reproducibility. The resin composition was optimized accordingly for the process to give R2. Since reduction of crosslinking moieties in the macromer results in low reproducibility of hydrogel layers, the radical mobility has to be decreased or the crosslinking has to be increased. Limitation of radical diffusion implies high macromer concentrations, which are difficult to process on resonator chips. To address this a recipe to combine high crosslinking during polymerization with low crosslinking of the final hydrogel was developed. Therefore, the hydrolysable bifunctional monomer MAAA was used in R2.

## 3. Results

The influence of laser settings was initially tested with the hydrogel resin R1. The macromer M2 that was used here was designed as a base for generic hydrogels to be used in a broader range of applications.

**Figure 4:**
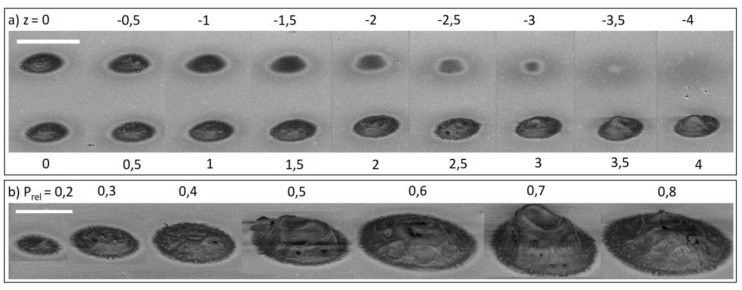
(**a**) Offset screening with values given in µm. Offset = 0 is at the automatic adjusted interface. At higher z-values the focus point moves into the surface. The scale bar is 5 µm; (**b**) screening of different P_rel_ settings ranging from 0.2 to 0.8. Scale bar is 3 µm. All samples were prepared on reflective chromium from R1. The interface (z = 0) was determined by the interferometry-based interface finder of the DLW system.

As can be seen from Figure 4, clear elliptical voxels known from non-hydrogel resins are not formed, but instead, a disc-like structure covers the surface. The influence of the z-alignment is of low influence for an offset value of larger than −0.5 µm as the screening has demonstrated. Lateral size of the dots does not increase, only the thickness increases slightly. Instead, the power setting is of high influence on polymer thickness and topology. P_rel_ in the range of 0.2 and 0.4 seems to be ideal for good polymerization control since exceeding this power range increases the dot little but instead results in central cavities that partially collapse. For lower power settings there is a threshold of P_rel_ = 0.2, meaning no polymerization is possible at lower values. At increasing z values, the size and quantity of hydrogel remains almost the same. We attribute this to absorption and scattering during polymerization, which results in higher polymerization rates at the side furthest from the surface. We believe that the geometry is determined primarily by diffusive and, at increasing laser power, thermal effects, resulting in the central cavities. We hypothesize that the polymerization starts inside the focus spot and the active chain ends diffuse outward. At higher powers, the center zone of the focus spot will heat up and increase the diffusion rate considerably, resulting in the central holes. As an indicator for this mechanism, we conducted experiments without a photoinitiator. No hydrogels were created unless the power was vastly increased to a point where the gels started boiling. In this case, only irregular hydrogel fragments could be seen in a large area around the focus zone. Preliminary experiments comparing macromers with non- crosslinked polymers in the solutions supported this theory, since the latter only resulted in a decreased polymerization windows (P_rel_ = 0.3–0.4), lower reproducibility and irregular structures.

The disc like shape is ideal for our application on the plate of the resonator, since this geometry poses far less resistance and thus hydrodynamic damping during resonator operation compared to an elliptical columnar voxel to surrounding water and can be created in one step over less than a second instead of scribing the whole disc. The resonators used have a different surface morphology compared to the smooth samples used in the preliminary experiments. This resulted in an uneven reflection and hotspots in the focus area, slightly decreasing the required power. Additionally, the gels used in the preliminary experiments were crosslinked to a degree that did not show sufficient pH dependent swelling to be detected. Therefore, the hydrogel composition was changed as described above, using R2.

**Figure 5:**
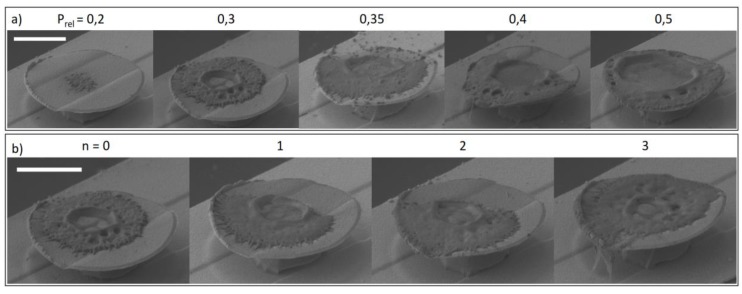
Power (**a**) and repetition (**b**) screening on resonators using R2. Power values are given in relative units and the repetitions are done at P_rel_ = 0.3. The number of repetitions is given by *n*. Each repetition is done at the same settings as the first spot and the time between each repetition was around 5 s to allow for heat dissipation and diffusion of reactive compounds. The scale bars are 3 µm.

As can be seen from Figure 5, the thickness and lateral size of the polymer disc is strongly dependent on the laser power and repetition steps with the same power setting. In several preliminary experiments, we realized that powers of P_rel_ = 0.2 to 0.4 gave the best results, meaning maximum coverage without overhanging gel. Overhanging hydrogel renders the resonator useless because meniscus formation fails and the complete resonator is wetted. Here, a power of P_rel_ = 0.3 seemed favorable, so it was used for the repetition experiments. Waiting times of around 5s between repetition steps can increase the polymer density and thickness with minor impacts on lateral distribution. After polymerization and development, the hydrogels were hydrolyzed in mild basic aqueous solutions (25 µM triethylamine) over night to achieve the desired pH responsiveness. Faster hydrolysis with high pH solutions is not recommended due to high swelling stress during the reaction and thus possible destruction of the gel.

To prove maintained responsivity, measurements in different pH solutions were conducted (Table 3) by covering the resonator under a 10 µL droplet. Under acidic conditions, the frequency dropped 20 kHz accompanied by an increased width of the resonant peak, similar to previous measurements [38]. This single sided wetting is metastable with the hydrogel on the resonator plate and drops within seconds to minutes to a broad, noisy peak, indicating a wetting of the resonator bridge. This allowed for measurements over several minutes during which time the peak did not shift. Adding one equivalent of the basic solution, the peak shifts 151 kHz within seconds and staying stable as in the previous measurement. The pH value of the mixture was expected to be at around 5, meaning it was higher than the gels swelling point [2]. Further addition of the base did not change the peak maximum considerably, as expected for the swelling curves plateau area. Under neutral and basic conditions, single sided wetting was impossible, implying a swelling of the gel over the sides of the plate or a change in wetting behavior.

**Figure 6:**
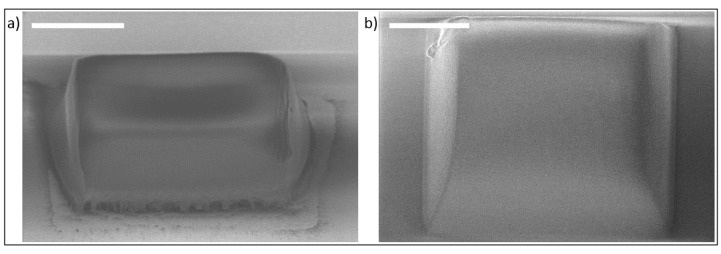
Blocks from solvent free R3; (**a**) on reflective surfaces (block measured at 9.33 µm × 22.8 µm) and (**b**) on transparent surfaces (block measured at 12.5 µm × 32.3 µm at the surface to 22.9 µm at the shrunk side). The laser scribe field is 30 µm × 30 µm × 10 µm. Scale bars are 10 µm.

The approach can be used to manufacture 3D structures as well, which we want to demonstrate in short by using macromer solutions with low amounts of large monomers (here: MMAES). After deposition onto the substrate, the solvent is removed by evaporation. Due to the size and geometry of the voxel, this protocol is not favored for our application here. Since diffusion is reduced vastly, it is possible to write 3D-structures like the example blocks shown in Figure 6. The edge rounding is likely due to shrinkage and proximity effects known from the literature [43,44]. Close to the reflective surface, the structure is highly porous and different from the bulk polymer. This is due to interference effects resulting in hot spots that can induce boiling, degassing and inhomogeneous polymer. On transparent surfaces, this effect is inexistent.

## 4. Conclusions

In summary, the application of a generic approach for structuring of a wide range of responsive, acrylate based hydrogels on a fragile, reflective microresonator MEMS device was demonstrated. Not only high precision but also minimal mechanical forces during structuring were proven by structuring gels on fragile structures. Performing resonance measurements in aqueous saline solutions in the fully wetted state gave proof of maintained sensing abilities. The use of macromers to limit diffusion gave control over structuring by limiting radical diffusion, resulting in lateral sizes of 2 to 5 µm and thicknesses of some hundred nanometers. Although the protocol was developed for thin layers, the applicability of the approach for 3D structures was demonstrated.

## Figures and Tables

**Table 1:** Molar ratios of monomer units in the different macromers.

Abbreviation	Crosslinker	MAA	NIPA
**M1**	1	1	
**M2**	1	6	4

Reaction conditions: Step 1: Monomer (7 mmol, half for MAAA respectively), AIBN (7 µmol), acetone (16 mL), 60 °C, 24 h. Step 2: Excess HEMA (2 mL), NEt_3_ (0.4 mL), DMAP (0.5 mg), 25 °C, 4 h.

**Table 2:** Compositions of hydrogel resin solutions.

Abbr.	Macromer	Macromer (mg)	BAPO (mg)	DMSO (µL)	MAA (µL)	HEMA (µL)	MAAA (µL)	MMAES (µL)
**R1**	**M1**	80	12	50	50	200		
**R2**	**M2**	90	12	100	300		300	
**R3**	**M1**	80	12					250

**Table 3:** Frequency shift of resonator under different conditions.

	Air	Citric acid ^a^	Citric acid ^b^	Na_2_HPO_4_^b^
Resonance frequency (kHz)	1939	1919	1120	969
Variance (kHz)	<1	n/a	17	16

Notes: Concentrations of citric acid 0.1 M (pH = 1.7) and Na_2_HPO_4_ 0.2 M (pH = 9) in 0.8 M aqueous sodium chloride solution for stable ion strength. Resonator bridge length is 125 µm. a) Partial wetting of the resonator is metastable and collapses within seconds to minutes to b) full immersion. Values of b) could be determined within a range of 2.5 kHz; pH value of mixed solution estimated at around 4.6–4.8. Hydrogels were made from R2 as described above (P_rel_ = 0.3, *n* = 0).
